# State of the art in the analysis of brominated flame retardants in biota and sediment: insights from the characterisation of two new certified reference materials

**DOI:** 10.1007/s11356-020-08950-7

**Published:** 2020-05-16

**Authors:** Marina Ricci, Penka Shegunova, Katrin Vorkamp

**Affiliations:** 1 European Commission, Joint Research Centre (JRC), Retieseweg 111, 2440 Geel, Belgium; 2 Aarhus University, Department of Environmental Science, Frederiksborgvej 399, 4000 Roskilde, Denmark

**Keywords:** Polybrominated diphenyl ethers (PBDEs), Hexabromocyclododecanes (HBCDDs), Fish, Quality assurance and control, Environmental monitoring, Food safety, Measurement uncertainty, Interlaboratory comparison

## Abstract

The Joint Research Centre (JRC) of the European Commission has recently released two new Certified Reference Materials (CRMs) for the analysis of brominated flame retardants (BFRs): the freshwater sediment ERM-CC537a and the fish tissue ERM-CE102. The production of these CRMs responds to the need of expanding the offer of quality assurance/quality control tools for the analysis of BFRs in the fields of environmental analysis and food control, especially for compliance purposes. The sediment ERM-CC537a carries certified values for polybrominated diphenyl ethers (PBDEs) and hexabromocyclododecane (HBCDD) diastereoisomers in the μg/kg range (dry mass), while the fish tissue ERM-CE102 is certified for PBDEs in the ng/kg range (wet weight). ERM-CC537a is the first reference material ever available with certified values for α-, β- and γ-HBCDD. The assignment of the certified values was performed via an intercomparison of expert laboratories. The evaluation of the data confirms the improving trend, observed in recent years, regarding the comparability of PBDE measurement results. The relative standard deviation (RSD) among laboratories is slightly better for the fish material (8–11%) compared to the sediment (9–15%), despite lower mass fractions in the biota matrix. The RSD of HBCDD data (17%) reveals that they are more challenging analytes. The average measurement uncertainty declared by the participants is about 30%, but an in-depth analysis of their performance reveals that it should be feasible to reduce the uncertainty budget.

**Electronic supplementary material:**

The online version of this article (10.1007/s11356-020-08950-7) contains supplementary material, which is available to authorized users.

## Introduction

Flame retardants are chemical additives used in a large variety of inflammable commercial products (e.g. electronic equipment, construction materials, furnishings and textiles), with the purpose of preventing the start and/or slowing the spread of fire. Polybrominated diphenyl ethers (PBDEs) and hexabromocyclododecanes (HBCDDs) belong to the additive-type brominated flame retardants (BFRs) which, due to the absence of covalent bonds to the polymer, are more easily released from the products than reactive flame retardants (Xu et al. 2009; Osako et al. 2004). PBDEs were produced in the three commercial mixtures Penta-, Octa- and DecaBDE. The production and use of all three technical products has been severely restricted in the EU since 2003 (EC 2003; EC 2002 repealed by EC 2011a). In addition, HBCDDs and DecaBDE are regulated under the EU Regulation Registration, Evaluation, Authorisation and Restriction of Chemicals (REACH) (EC 2006; EC 2017a). Penta- and OctaBDE, HBCDDs and DecaBDE were added to the Stockholm Convention on Persistent Organic Pollutants in 2009, 2013 and 2017, respectively (UN, Stockholm Convention http://www.pops.int, Accessed 6 April 2020). Despite their restrictions and bans, BFRs continue to remain ubiquitous in the environment, owing to their persistence, long-range atmospheric transport (Okonski et al. 2014), bioaccumulation and biomagnification (HELCOM 2018; Ma et al. 2013). Due to their high octanol-water partition coefficients (log K_ow_) (Braekevelt et al. 2003), they typically adsorb onto suspended and bed sediments and partition into lipids in organisms (Wenning et al. 2011; Zegers et al. 2003; de Boer et al. 2003). Exposure to BFRs, both via the environment, the food chain and from indoor sources, is a risk for human health. Besides the recognised endocrine-disruptive effects of the PBDEs, evidences on neurodevelopmental toxicity and indications of cancer have emerged (Lilienthal et al. 2006; Hoffman et al. 2017). Toxic effects in animals have been found for HBCDDs (Guo et al. 2019; Lyche et al. 2015).

The monitoring of BFRs in environmental and food samples is an ongoing task for analytical laboratories all over the world. In Europe, PBDEs and HBCDDs are included in the list of priority substances of the EU Water Framework Directive (WFD) with defined environmental quality standards (EQS) in water and biota (EC 2013). They also have to be monitored in fish and seafood (among other foodstuff) following the Commission Recommendation 2014/118/EU (EC 2014). Monitoring of BFRs in biota and sediment is also foreseen in other programmes, for example the Coordinated Environmental Monitoring Programme (CEMP) of the Oslo-Paris Commission (OSPAR) or the Arctic Monitoring and Assessment Programme (AMAP). The reliability of measurement results is imperative, especially for compliance purposes. For control and monitoring laboratories, the applied analytical methodologies should be validated in accordance with the requirements listed in ISO/IEC 17025 or similar standards. The analysis of certified reference materials (CRMs) is an essential component in this context, both in environmental and food-related legislation, and recommended in the light of ensuring quality and comparability of data (EC 2009; EC 2017b).

This study presents the development of two CRMs recently released by the Joint Research Centre (JRC) of the European Commission in Geel, Belgium. The freshwater sediment ERM-CC537a, certified for PBDEs and HBCDDs, and the fish tissue ERM-CE102, certified for PBDEs only, were both prepared from naturally contaminated material to ensure their commutability and suitability for routine analyses. The commutability (or similarity of analytical behaviour to routine samples) is particularly pronounced in the case of ERM-CE102, whose matrix is a wet paste. In addition, the certified values are assigned relative to wet weight, making its use for compliance check towards legal limits established on a wet weight basis (e.g. WFD EQS, limit of quantification in the food control sector) more straightforward and possibly less biased than in the case of freeze-dried materials. The primary aim of this study was the assignment of certified values. However, it also presents interesting insights into the achievable analytical quality of state-of-the-art PBDE and HBCDD analysis conducted by expert laboratories in charge of routine measurements in the field of food control and environmental monitoring. In this frame, a more extensive discussion targeting the evaluation of the measurement uncertainty is presented.

## Materials and methods

### Sampling and processing of the CRMs

#### ERM-CC537a

Freshwater sediment was sampled in November 2011 from a small Belgian river included in the Flemish sediment monitoring network (VMM, Vlaamse Milieumaatschappij https://www.vmm.be, Accessed 6 April 2020). About 700 kg of top layer sediment (about 20 cm depth) was collected in high-density polyethylene containers and transported to the JRC processing laboratories in Geel.

**Fig. 1:**
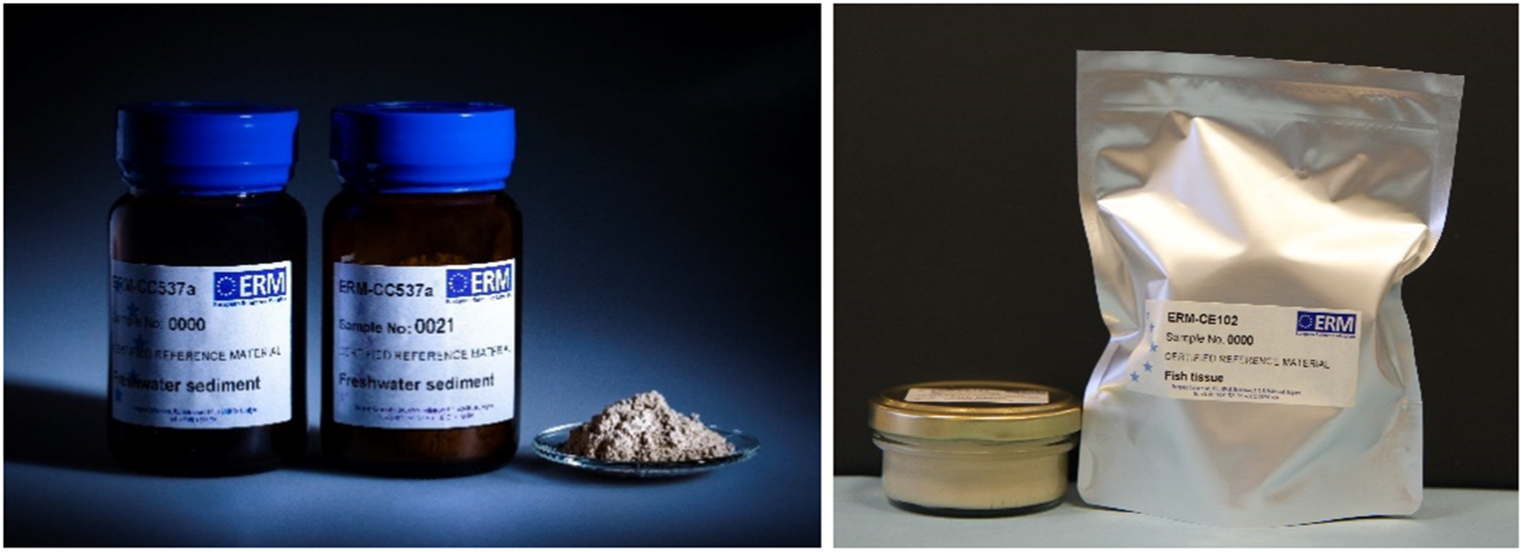
ERM-CC537a (left) and ERM-CE102 (right) CRM units

The wet sediment was subjected to air-drying at 35 °C for about 1 week in ventilated drying cabinets (Hereaus, model UT 6760, Langenselbold, DE). Subsequently, the sediment was manually crushed and sieved over a 1-mm stainless steel sieve (Russel Finex, London, UK). After about 1 month of storage at room temperature, the bulk sediment was jet-milled (Alpine, Augsburg, DE) and further sieved using a 125-μm stainless steel sieve (Russel Finex, London, UK). The homogenisation was carried out using a Dynamix-200 CM mixer (WAB, CH) for about 2 h and finally the sediment was dispensed through a cone mixer in portions of about 40 g into 60-mL amber glass bottles with screw caps having an aluminium disc as insert (Fig. 1). Sterilisation of the material was performed by γ-irradiation using a dose between 7 and 12.5 kGy. Afterwards, ERM-CC537a was stored at + 4 °C awaiting further tests. A total of 1500 units of ERM-CC537a were produced.

#### ERM-CE102

Two fish species were employed as starting materials, rainbow trout (*Oncorhynchus mykiss*) locally sourced from a Belgian aquaculture farm, and wild Wels catfish (*Silurus glanis*) originating from the area of the Flix reservoir of the Ebro river (Spain), caught in spring 2011 and stored frozen at the JRC laboratory. Both starting fish materials were analysed for BFRs levels prior to CRM preparation: BFRs were detected in the catfish, but not in the trout. Thus, the mass fractions in the catfish served as the basis for the processing scheme and, in order to reach the desired BFRs levels in the candidate CRM, the two fish species were mixed according to a mass ratio trout:catfish = 98:2.

About 100 kg of trout and about 1.8 kg of catfish were sliced into fillets (removing bones and skin). The fillets were cut in small cubes, shock-frozen in liquid nitrogen and cryogenically milled using a Palla VM-KT cryogenic vibrating mill (KHD, Köln, DE), separately for the two species. The resulting pastes were first stored at − 20 °C and then allowed to thaw at + 4 °C, before pre-cooking in glass jars at 85 °C in an autoclave JBTC AR092 pilot retort (JBT, Sint-Niklaas, BE). After this pre-heating step, the catfish and trout batches were first separately homogenised in a Stephan UM12 mixer (Hameln, DE), followed by a step-wise mixing scheme using a Stephan UM200 mixer, during which the catfish batch was diluted with batches of blank trout until obtaining a well homogenised fish paste. Approximately 40 g of the fish paste was filled into 60-mL glass jars using a Unifiller machine (Lörrach, DE). The jars were closed with twist-off lids using a Lenssen twist-off machine (Sevenum, NL) inside a chamber filled with steam. The under-pressure created in the head-space over the paste after cooling down ensures that the sensor of the lid remains pressed-down as long as the seal is not broken. Upon opening, the click of the lid will indicate that the sample is not compromised. The filled jars were sterilised in an autoclave at 121 °C (JBT, Sint-Niklaas, BE). After labelling, each jar was placed into a pre-labelled multilayer pouch which was thermo-sealed using a DAKLA sealing machine (Daklapak, Kortrijk, BE) and stored at + 4 °C awaiting further tests (Fig. 1). Approximately 1400 units of ERM-CE102 were produced.

### Homogeneity and stability of the CRMs

#### Homogeneity

A key requirement for any reference material aliquoted in units is the equivalence between those units, i.e. ensuring that the certified values of the CRM are valid for all units, within the stated uncertainties. ISO 17034 requires RM producers to quantify the between-unit variation. Quantification of between-unit inhomogeneity (as standard deviation, to be introduced as a component in the final certified uncertainty) was carried out by analysis of variance (ANOVA) on a number of units selected using a random stratified sampling scheme covering the whole batch. Besides the between-unit homogeneity, quantification of within-unit inhomogeneity (i.e. microhomogeneity) is necessary to determine the minimum sample intake of the CRM. The within-unit inhomogeneity was established by comparison of variances obtained using the F-test for equality of two-sample variances at a confidence level of 95%.

##### ERM-CC537a

Ten bottles of ERM-CC537a were selected. Four independent samples of at least 750 mg were analysed from each selected unit under repeatability conditions by gas chromatography with electron capture negative ionization low resolution mass spectrometry (GC-ECNI-LRMS) for BDE28, 47, 99, 100, 153, 154, 183 and 209 and by high-performance liquid chromatography with tandem mass spectrometry (HPLC-MS/MS) for HBCDDs. The data were evaluated with respect to analytical trends (and corrected when needed) and for outliers (only excluded in case of technical reasons). For the microhomogeneity, a minimum of eight independent replicate analyses were performed per sample intake for the following decreasing sample intakes: 750 mg, 500 mg and 200 mg.

##### ERM-CE102

Between eight and sixteen units were selected. Three independent samples from each selected unit were analysed for BDE28, 47, 49, 99, 100, 153, 154, 183 and 209 by GC with high-resolution mass spectrometry (GC-HRMS). The sample preparations had to be split over several days; therefore, day-to-day effects were addressed and data normalised, if needed, to detect the presence of trends and outliers. In the case of BDE49, an outlier was detected and the between-unit inhomogeneity was modelled as a rectangular distribution limited by the outlying unit mean. For the microhomogeneity, nine independent replicates were analysed per sample intake for the following decreasing sample intakes: 8 g, 5 g and 2 g.

#### Stability

Stability testing is necessary to establish the conditions for storage (long-term stability) as well as the conditions for dispatch of the materials to the customers (short-term stability). The influences of time and temperature were regarded as relevant for these CRMs and the stability studies were carried out using an isochronous design (Lamberty et al. 1998). After evaluation for analytical trends and outliers, the data were plotted as mass fraction against storage time and the slopes of the obtained regression lines were tested for statistical significance, evidencing potential increasing/decreasing trends of the target analytes over time. The uncertainties of stability during dispatch and storage (to be included in the final uncertainty budget of the certified value) were estimated as described in Linsinger et al. (Linsinger et al. 2001).

##### ERM-CC537a

For the short-term stability study, samples were stored at + 18 °C and + 60 °C for 0, 1, 2 and 4 weeks (reference temperature set to − 20 °C). From each of the two units selected per storage time, three sub-samples were analysed by GC-HRMS for PBDEs (see sub-section "Homogeneity") and ultra-performance liquid chromatography with tandem mass spectrometry (UPLC-MS/MS) for HBCDDs. For none of the parameters, trends were statistically significant (95% confidence level) at + 18 °C. However, the slopes of the regression lines were significantly different from zero on at least a 95% confidence level at + 60 °C for BDE28, 47, 153, 154, 183, 209, α- and γ-HBCDD. ERM-CC537a must be therefore shipped under cooled conditions (not exceeding + 18 °C).

For the long-term stability study, samples were stored at + 4 °C and + 18 °C for 0, 8, 16 and 24 months (reference temperature set to − 20 °C). From each of the two units selected per storage time, three sub-samples were analysed by GC-ECNI-LRMS for PBDEs and HPLC-MS/MS for HBCDDs. No significant trend was detected for any of the analytes (95% confidence level), except for β-HBCDD at + 4 °C (but not at + 18 °C). ERM-CC537a will therefore be stored at + 18 °C.

##### ERM-CE102

For the short-term stability study, samples were stored at + 18 °C and + 60 °C for 0, 1, 2 and 4 weeks (reference temperature set to +4 °C). From each of the two units selected per storage time, three sub-samples were analysed by GC-HRMS for BDE28, 47, 99, 100, 153, 154, 183, 209). For none of the parameters, trends were statistically significant (95% confidence level) at + 18 °C. A statistically significant positive trend (95% confidence level) was observed for BDE154 at + 60 °C. This was regarded as a statistical artefact because other possible reasons were ruled out, such as degradation of the matrix (which would result in a positive trend for all PBDEs, which was not the case) or dehalogenation of BDE209 (no decreasing trend was observed in the mass fraction of this congener). However, a conservative approach was chosen and it was decided to ship the material under cooled conditions (not exceeding + 18 °C).

For the long-term stability study, samples were stored at + 4 °C and + 18 °C (reference temperatures set to − 20 °C and + 4 °C, respectively) for 0, 8, 16 and 24 months. From each of the two units selected per storage time, three sub-samples were analysed by GC-HRMS for PBDEs. No significant trend was detected for any of the analytes (95% confidence level) at + 4 °C, while at + 18 °C, positive significant trends (regarded as statistical artefacts) were detected for BDE49 and BDE183. ERM-CE102 will therefore be stored at + 4 °C.

### Characterisation by interlaboratory comparison

#### Design of the characterisation studies

The material characterisation, i.e. the process of determining the property values of a reference material, was based on an interlaboratory comparison of expert laboratories applying different measurement procedures to demonstrate the absence of a measurement bias. The interlaboratory comparisons took place between November 2016 and March 2017 for ERM-CC537a and from September to December 2018 for ERM-CE102. The laboratories were selected based on criteria that comprised both technical competence and quality management aspects. Each participant was required to operate a quality system and to deliver documented evidence of its proficiency in the analysis of PBDEs and HBCDDs in sediment (for ERM-CC537a) and PBDEs in biota (for ERM-CE102) or similar matrices, by submitting results of interlaboratory comparison exercises and/or method validation data. Having a formal accreditation was not mandatory, but meeting the requirements of ISO/IEC 17025 was obligatory. The participation required the application of validated methods.

The analysis protocol to be respected in the characterisation study included the following points: (1) sample preparations and measurements had to be spread over at least 2 days to ensure intermediate precision conditions of analysis, (2) fresh calibration solutions had to be prepared for each day of measurement, (3) each participant had to analyse a sample of NIST SRM 1944 New York/New Jersey Waterway Sediment (in the case of ERM-CC537a) and NIST SRM 1946 Lake Superior Fish Tissue (in the case of ERM-CE102) as a blind method quality control sample alongside the CRM samples.

Laboratories were also requested to give estimations of the expanded uncertainties of the mean value of the measurement results. No approach for the estimation was prescribed, i.e. top-down and bottom-up approaches were regarded as equally valid procedures.

##### ERM-CC537a

Thirteen laboratories (for a total of fourteen datasets for PBDEs, one laboratory providing two datasets, and nine datasets for the HBCDDs) were selected. For each dataset, laboratories received two units of ERM-CC537a and had to report six independent results (three per unit) for PBDEs and HBCDD isomers on a dry mass basis. The water and volatiles’ content had to be determined on each unit in duplicate (according to a prescribed oven-drying procedure).

##### ERM-CE102

Twelve laboratories (for a total of fourteen datasets, two laboratories providing two datasets) were selected. For each dataset, laboratories received two or three units of ERM-CE102 and had to report six independent results (three or two per unit, respectively) relative to wet weight.

#### Analytical methods

##### ERM-CC537a

A variety of extraction procedures [e.g. Soxhlet, accelerated solvent extraction (ASE) and solid-phase extraction (SPE)] and clean-up methods [e.g. alumina and acidic silica gel columns, gel permeation chromatography (GPC)] with different instrumental determination techniques (GC-LRMS, GC-HRMS, GC-MS/MS, HPLC-MS/MS, UPLC-MS/MS) were applied by the participants (Tables S1 and S2 in Online Resource).

Two analytical methods for the analysis of PBDEs in sediment [(GC-ECNI-MS and GC with electron ionization-isotope dilution tandem MS (GC-EI-IDMS/MS)] were validated in-house at the JRC for participating in the characterisation study of ERM-CC537a (L12S and L13S in Table S1, Online Resource). The sample preparation applied was the same in both methods: 2 g of sediment was mixed with approximately 5 g of a Cu/Na_2_SO_4_ mixture (1/3, w/w), loaded in an 11-mL ASE thimble (containing a filter at the bottom) and spiked with 50 μL (by weight) of the internal standard solution. The extraction was carried out with an ASE 200 system (Dionex™, Thermo Scientific™, Sunnyvale, CA, USA), applying the following conditions: pressure 1500 psi, temperature 120 °C, pre-heating time 5 min, heating time 6 min, static time 10 min, flush volume 150%, purge time 120 s, static cycles 3, solvent hexane/acetone (1/1). The extract was dried over Na_2_SO_4_ and evaporated til ~ 1 mL. Further clean-up was performed by SPE (Gilson SPE-GX-274ASREC™, Middleton, WI, USA) using a Bond Elute PCB cartridge (1 g, 3 mL, ChromTech, Apple Valley, MN, USA), eluting with 4 mL of hexane, (flow speed ~ 1 mL/min). Before instrumental analysis (details reported in the Online Resource), the extract was concentrated under a gentle steam of nitrogen (VLM EC1/VLM Eva2–Nitrogen concentrator).

##### ERM-CE102

Several extraction procedures [e.g. ASE, Soxhlet, organic solvent(s) extraction, liquid-liquid extraction (LLE)] and clean-up methods [e.g. carbon, alumina and multilayer (acidic, basic and neutral) silica gel columns, GPC and a combination thereof] with different instrumental determination techniques [GC-LRMS both in EI and ECNI modes, GC-HRMS and GC-MS/MS in EI mode] were applied (Table S3 in Online Resource).

#### Technical and statistical evaluation of the characterisation results

The technical evaluation of the submitted datasets included (1) compliance to the analysis protocol, (2) critical screening of values reported as below limit of detection (LOD) or limit of quantification (LOQ), (3) agreement of the measurement results with the assigned values of the blind quality control samples (NIST SRMs) and (4) coherence of the repeatability evinced from the characterisation dataset with the one declared by the laboratory according to their method validation. Based on these criteria, between a minimum of two and a maximum of six non-compliant datasets were excluded from the value assignment of certified properties.

For the statistical evaluation, results were tested for normality, outlying means (Grubbs test) and outlying standard deviations (Cochran test) at a 99% confidence level. Standard deviations within and between laboratories were calculated using one-way ANOVA.

All data and information related to the certification of ERM-CC537a and ERM-CE102 are available in the respective certification reports and certificates, to be found at https://crm.jrc.ec.europa.eu/.

## Results and discussion

### CRMs characterisation and fitness for purpose

The homogeneity and stability of ERM-CC537a and ERM-CE102 were ascertained as fit for purpose (Tables S4 and S5 in Online Resource).

**Fig. 2:**
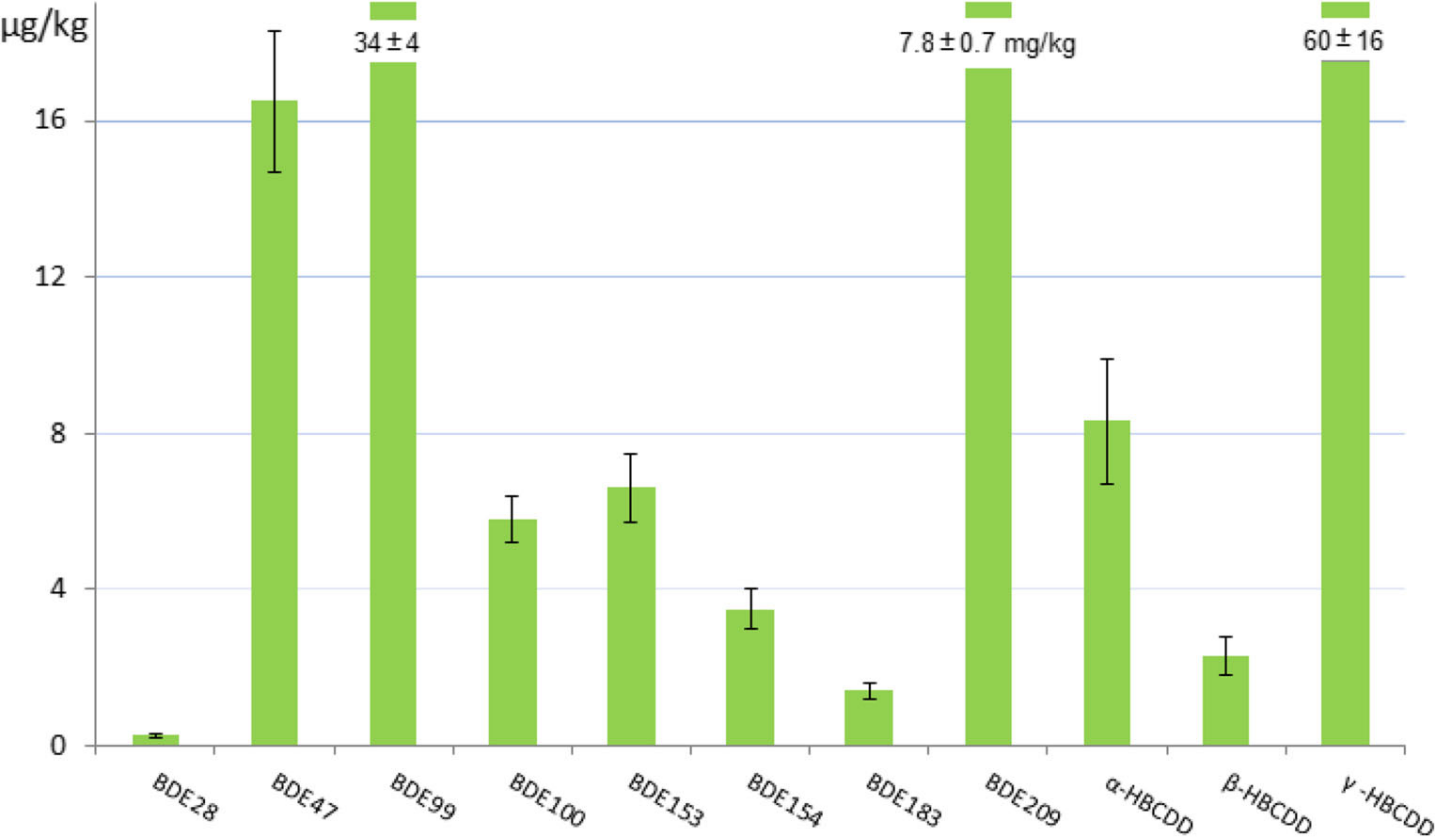
PBDEs and HBCDDs in ERM-CC537a: certified values and uncertainties expressed in μg/kg (in mg/kg for BDE209)

**Fig. 3:**
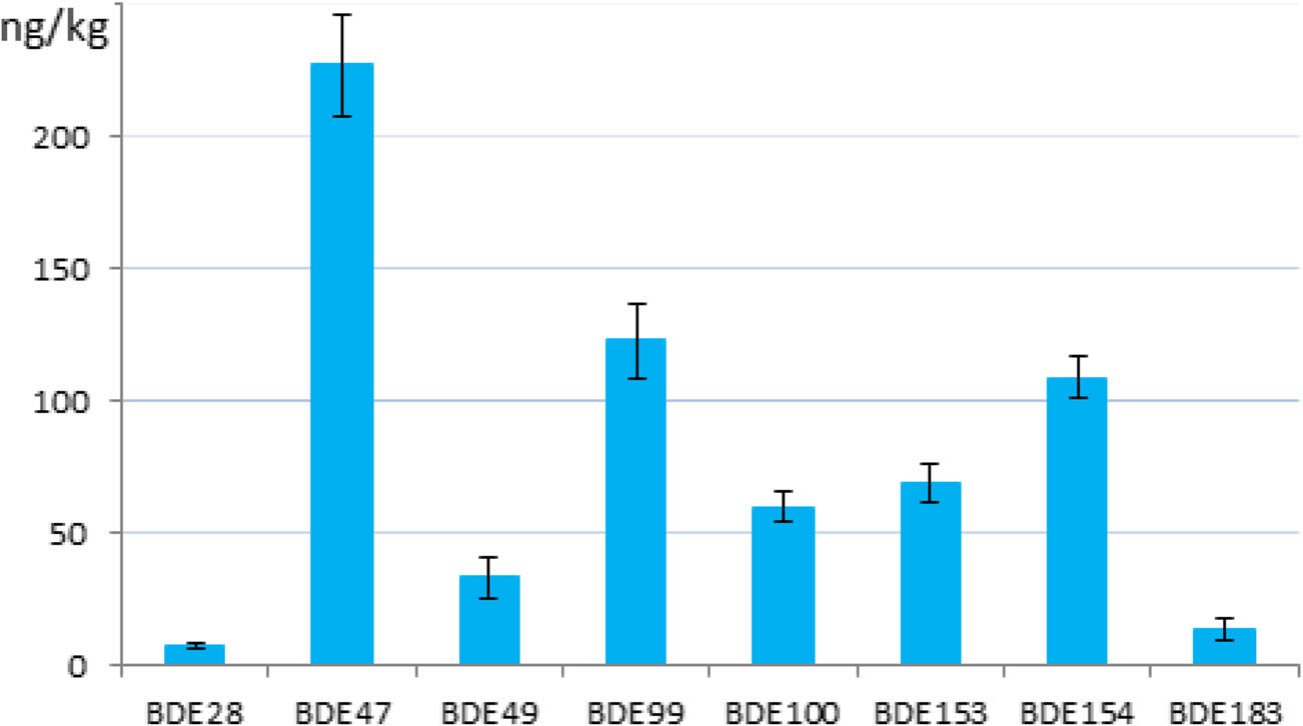
PBDEs in ERM-CE102: certified/indicative values and uncertainties (converted to ng/kg for a clearer view of the data)

The unweighted means of the means of the accepted datasets were assigned as certified value for each parameter (Figs. 2 and 3, Table S6 in Online Resource). The uncertainties of the certified values are composed by contributions related to the characterisation by the expert laboratories, potential between-unit inhomogeneity, potential degradation during transport and long-term storage and expanded by an appropriate coverage factor k (the different contributions are summarised in Tables S4, S5 and S7 in Online Resource).

The certified values of the PBDEs and HBCDDs in the freshwater sediment ERM-CC537a are in the range of μg/kg except for BDE209 (7.8 mg/kg) and reflect well the levels generally found in similar matrices in the environment although regional differences may exist (Law et al. 2008; Yogui and Sericano 2009; Hong et al. 2010; Ramu et al. 2010; Law et al. 2014; Rüdel et al. 2017).

The ∑PBDEs (28, 47, 99, 100, 153, 154, 183) in ERM-CC537a amounts to 68.09 μg/kg and rises up to 7868.09 μg/kg (dry mass) when BDE209 is included, which indicates a relatively highly contaminated site in Belgium, as informed by the Flemish sediment monitoring network (VMM, Vlaamse Milieumaatschappij https://www.vmm.be, Accessed 6 April 2020), before the sampling for the CRM was carried out. Heavily industrialised areas can show even higher levels of contamination, with value of ΣPBDEs up to 14,000 μg/kg (Eljarrat et al. 2007).

The distribution pattern of the PBDEs in ERM-CC537a (Fig. 2) evidences the dominance of BDE47 and BDE99 over the other lower brominated congeners, and the heavy presence of BDE209. This could indicate a past more extensive use of the PentaBDE and DecaBDE technical mixtures in comparison to the OctaBDE in the area of the CRM sampling. Similar PBDEs patterns can be found in reports on PBDE contamination in the European environment but also elsewhere (Law et al. 2008; Yogui and Sericano 2009; Hellar-Kihampa 2013; Song et al. 2004; Verhaert et al. 2013).

ERM-CC537a presents similar PBDEs levels (mostly within a factor of 10) to NIST SRM 1944 New York/New Jersey Waterway Sediment, with the exception of BDE209, which is 100 times higher in ERM-CC537a than in SRM 1944. It is worth to note that NIST SRM 1944 carries only reference (and not certified) values for this class of brominated flame retardants (Ricci et al. 2016).

The ∑HBCDDs in ERM-CC537a, amounting to 70.6 μg/kg dry mass, additionally confirms the sampling site as being relatively contaminated when compared to literature records from Europe and Asia (Rüdel et al. 2017; Law et al. 2006; Wang et al. 2017; Zhang et al. 2018). The relative amounts of the three diastereoisomers translate into approximately 12%, 3% and 85% for α-, β-, and γ-HBCDD, respectively, and correspond well to what is generally found in most suspended particular matters and sediments (and similar to commercial HBCDD formulations) (Rüdel et al. 2017). It is worth to mention that ERM-CC537a is the first ever RM carrying certified values for the HBCDD diastereoisomers (e.g. in NIST SRM 1944 New York/New Jersey waterway sediment, α-, β- and γ-HBCDD have indicative values without assigned uncertainty).

ERM-CE102 is certified for PBDE mass fractions in the range of ng/kg, with a total of 647.7 ng/kg wet weight for ∑PBDEs (28, 47, 49, 99, 100, 153, 154, 183), more matching the lower end of the contamination spectrum reported in the literature for fish (EC 2011; Eljarrat and Barceló 2018; Chen et al. 2013). ERM-CE102 complements the availability of fish CRMs for PBDE analysis by providing a material with certified values at mass fractions roughly hundred times lower than the NIST SRM 1946 (Lake Superior Fish Tissue) and SRM 1947 (Lake Michigan fish tissue). In addition, while the NIST SRMs are deep-frozen materials that need to be stored at − 80 °C, the ERM-CE102 is a fish paste to be stored at + 4 °C (Poster et al. 2003). The PBDE quantities in ERM-CE102 were obtained by mixing two fish starting materials (one contaminated and one ‘blank’) and they mirror naturally occurring levels, while not representing one particular species or location. The assignment of a certified value for BDE138 was not possible because all measurement results were below the LOD.

An EQS of 0.0085 μg/kg wet weight was set for biota (fish) under the WFD in 2013 (EC 2013) for the sum of BDEs 28, 47, 99, 100, 153 and 154, while an LOQ of 0.01 μg/kg wet weight is required by the 2014 Commission Recommendation (EC 2014) for the analytical methods employed in the monitoring of BDEs 28, 47, 49, 99, 100, 138, 153, 154, 183 and 209 in fish (among other food commodities). These values are very low and challenging, even for the most advanced analytical chemistry techniques to date. The assignment of a certified value at this mass fraction level is still an unmet achievement and, in the view of the authors, presently unreachable, because the related uncertainty would become extremely large, undermining the usefulness of the certified value. In ERM-CE102, the WFD ∑PBDEs (28, 47, 99, 100, 153, 154) amounts to 0.5977 μg/kg wet weight, i.e. almost two orders of magnitude higher than the EQS, notwithstanding the lowering of the natural contamination levels occurring in the processing of this CRM. The certification of BDE28 at a mass fraction of 0.0077 μg/kg, thus matching the EQS, was so difficult that only an indicative value could be assigned. The applicability of the PBDEs EQS value, calculated to protect human consumers based on observed effects of one single congener (BDE99) on rats and including very large safety factors (Yang et al. 2015), is an ongoing subject of discussion by the monitoring laboratories community. The reality sees much higher levels of PBDEs present in the environment, often 100 to 1000 times the EQS (EC 2011b). In this respect, ERM-CE102 is to be considered as an appropriate reference material to be used as a quality control tool in the routine monitoring of environmental samples.

The levels of the individual PBDEs in ERM-CE102 match those reported in the literature (Fig. 3): the dominance of BDE47 followed by BDE99 is consistent with the general pattern in biota samples, confirming the commutability of the CRM (Verhaert et al. 2013; Yang et al. 2015; Miege et al. 2012; Peng et al. 2007). In these references, the average ratio BDE47/BDE99 in biotic sample is generally between 3 and 6, while in ERM-CE102, it is 1.8. It is well-known that reductive debromination is a significant degradation mechanism for BDE99, 183 and 209 in certain fish species. A significant amount of BDE99 is transformed in the gut of common carp to BDE47, readily assimilated in the fish tissues (Stapleton et al. 2006; Stapleton et al. 2004). One of the initially targeted PBDEs for certification in ERM-CE102 was BDE209. Unfortunately, unsatisfactory results obtained during the certification process, especially with regard to the homogeneity evaluation (Table S4 in Online Resource), brought us to the decision of not proceeding with the assignment of a certified value for this congener (more details are available in the certification report). Nevertheless, the mass fraction of BDE209 was repeatedly measured in several occasions, including the homogeneity and stability studies, and reported as approximately 600 ng/kg (wet weight). The presence of a relatively high level of BDE209 was surprising, especially taking into account the ‘dilution’ of the catfish with blank trout during the processing of the CRM. BDE209 is generally present at much lower mass fractions in fish because of debromination mechanisms and possibly lower bioavailability (Miege et al. 2012; Viganò et al. 2011; Noyes et al. 2011; Pulkrabová et al. 2007). The BDE209 mass fraction measured in ERM-CE102 might be an indication of an exceptionally heavy BDE209 pollution in the Flix reservoir of the Ebro river where the catfish was collected (Eljarrat and Barceló 2008). Polder et al. (Polder et al. 2014) also reported high mass fractions of BDE209 in tilapia fish (168 ng/g lipid weight) from Lake Victoria in Tanzania, possibly explained by a constant release of this congener into the water of the lake by nearby industrial areas.

The successful outcome of the characterisation study additionally confirms the commutability, i.e. comparability of analytical behaviour to ‘real world’ samples, of these CRMs via the agreement of results obtained from measurement procedures based on different principles and routinely applied for the analysis of BFRs in environmental and food samples. In particular for ERM-CE102, the commutability to fresh routine samples is enhanced by its consistency as wet paste: measurement results can be directly compared to EQS values (expressed as wet weight) avoiding the application of dry/wet mass fraction conversion factors.

### Interlaboratory comparability

Following the first reports on interlaboratory comparability on PBDEs (de Boer and Cofino 2002; de Boer and Wells 2006), a few other studies have been published presenting results from interlaboratory comparisons for BFRs in solution, sediment (or similar matrix) and biota (Bremnes et al. 2019). The agreement among laboratories seems to have improved over time, especially for BDE209, reaching coefficients of variation (CVs) of 20% and less (Duffek et al. 2008). For HBCDDs, a different situation must be flagged with a recent intercomparison on test solution showing CVs > 50% (Melymuk et al. 2015).

The analysis and comparison of the characterisation datasets of ERM-CC537a and ERM-CE102 provide interesting insights in the present state-of-the-art for the determination of PBDEs and HBCDDs in environmental samples and confirms the improving trend mentioned previously.

Considering the set of laboratories whose measurements were used in the assignment of the PBDE values, the relative standard deviation (RSD) among the laboratories in ERM-CC537a ranged between 9 and 15% (*n* = number of datasets between 9 and 12, with the exception of *n* = 6 for BDE209) while for ERM-CE102, the RSD ranged between 8 and 11% (5 < *n* < 11) (Table 1). A higher RSD of 18% was observed for BDE28 in the sediment sample (present at a very low mass fraction, close to the LOQ for some laboratories, see Table S1 and Table S6 in Online Resource) and BDE183 in the fish sample (only 4 datasets valid for value assignment, Table 1). The narrow range of RSDs obtained among datasets confirms the importance of selecting only expert laboratories meeting the requirements of ISO/IEC 17025 and exclusively applying validated methods. The overall performance of the laboratories seems slightly better in the case of the fish than of the sediment sample. One would expect a more difficult clean-up in the case of the fatty biota rich in co-extractives (lipid content of 6.9 m/m %) in comparison with an abiotic sample. In addition, the fish material was characterised by much lower PBDE mass fractions than the sediment (difference of two orders of magnitude for the certified PBDEs, Table S6 in Online Resource). However, sulphur, other sediment components and a generally more massive presence of additional organic pollutants could also interfere with the BFR determination in sediments (Webster et al. 2009).

**Table 1:** Relative standard deviation (RSD) among laboratories in the certification exercises of ERM-CC537a and ERM-CE102

	RSD among laboratories %	
	ERM-CC537a Freshwater sediment	ERM-CE102 Fish tissue
BDE28	18 (*n* = 11)	11 (*n = 5*)
BDE47	13 (*n* = 10)	9 (*n* = 8)
BDE49	–	11 (*n* = 7)
BDE99	12 (*n* = 11)	11 (*n* = 11)
BDE100	9 (*n* = 10)	11 (*n* = 10)
BDE153	15 (*n* = 12)	8 (*n* = 10)
BDE154	12 (*n* = 9)	8 (*n* = 9)
BDE183	12 (*n* = 9)	18 (*n* = *4*)
BDE209	9 (*n* = 6)	–
α-HBCD	16 (*n* = 6)	–
β-HBCD	16 (*n* = 6)	–
γ-HBCD	18 (*n* = 5)	–

*n*, number of datasets

The average RSD among laboratories observed for the analysis of HBCDD diastereoisomers in the sediment, ≈ 17%, seems to confirm that they are presently still more challenging analytes than the PBDEs. This is also indicated by the lower number of laboratories available for the certification of HBCDDs which mirrors the lower number of valid datasets used in the value assignment.

The satisfactory RSDs obtained in the interlaboratory studies for the PBDEs helped in keeping the uncertainty contribution of the characterisation (estimated as the relative standard error of the laboratories’ mean) for both CRMs down to an average of ≤ 4% (Table S7 in Online Resource). This contributed to the successful outcome of the PBDEs certification for both CRMs, with average expanded certified uncertainties of 12.8% for ERM-CC537a and 11.5% for ERM-CE102, respectively. The results are different for the HBCDD diastereoisomers, for which the average certified uncertainty was 22.6%, signalling greater difficulty of certification for these compounds.

The uncertainties of the certified values are, for all PBDE congeners, lower in ERM-CE102 than in ERM-CC537a. Considering the pool of PBDE congeners certified in both materials (47, 99, 100, 153, 154), the average certified uncertainty is 9.6% for ERM-CE102 against 12.1% for ERM-CC537a (Table S6 in Online Resource). The cause of this difference is to be found in the higher uncertainty contribution related to homogeneity for ERM-CC537a compared to ERM-CE102, an average of 2.5% against 0.9%, while the average uncertainty contributions related to long-term stability and characterisation show very similar or even equal values for both materials (Tables S4 and S5 in Online Resource). The higher uncertainty contribution related to homogeneity in the case of the sediment ERM-CC537a can be attributed to the higher variability in relation to repeatability of the analytical method applied in the homogeneity study, compared to the one used for the fish ERM-CE102, rather than indicating a real worse homogeneity of the material. The average RSD of the measurements for the homogeneity (considering the pool of PBDEs certified in both CRMs) equals to 5.6% for the ERM-CC537a against 1.2% for the ERM-CE102.

### Measurement uncertainty

#### Measurement uncertainty evaluation

The evaluation of the measurement expanded uncertainties (U) reported by the laboratories for the PBDEs reveals a wide range, depending on the specific congener, ranging from 10 up to 50%, and in very few cases even above. The average U is slightly lower in the case of the certification of ERM-CE102 compared to ERM-CC537a, 26% and 30%, respectively (Table 2). The average U for the HBCDD diastereoisomers is also very similar, 31% (all participant laboratories applied either HPLC or UPLC in combination with ESI negative MS/MS detection).

**Table 2:** Average measurement uncertainty (calculated from the laboratories’ reports) in the certification exercises of ERM-CC537a and ERM-CE102

	Average measurement uncertainty %	
	ERM-CC537a Freshwater sediment	ERM-CE102 Fish tissue
BDE28	26	33
BDE47	29	27
BDE49	–	24
BDE99	27	26
BDE100	28	23
BDE153	27	21
BDE154	32	24
BDE183	32	33
BDE209	35	–
α-HBCD	32	–
β-HBCD	31	–
γ-HBCD	30	–

**Fig. 4:**
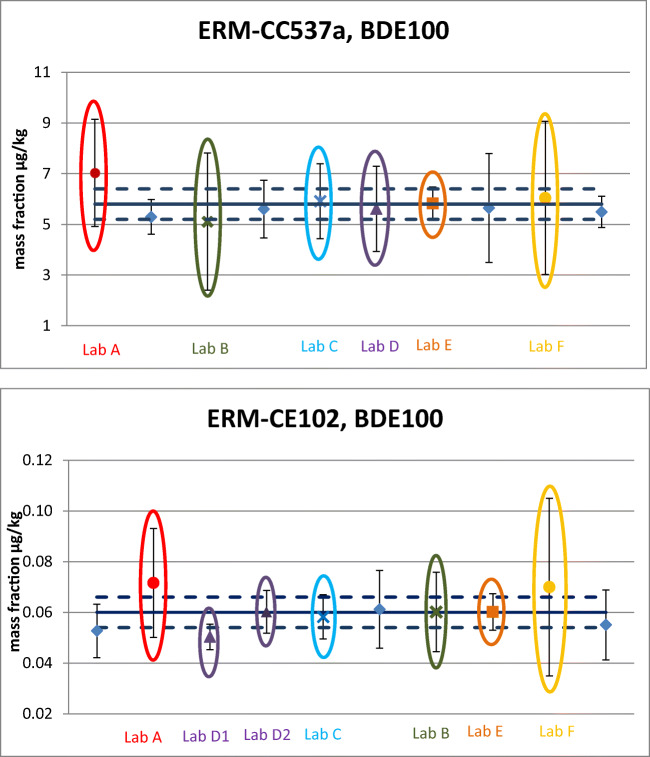
Comparison of reported measurement uncertainties for BDE100 in the characterisation study for ERM-CC537a (top) and ERM-CE102 (bottom); certified value (solid line) ± expanded uncertainty (dashed lines)

The measurement uncertainties reported by some laboratories are very large and seem to be overestimated, based on the RSDs of the characterisation datasets. For example, the RSDs (calculated from six independent results) of the datasets submitted by Laboratory A were 6 and 14% for ERM-CC537a and ERM-CE102, respectively, and 9% and 7% for Laboratory F, against a reported measurement uncertainty of 30 and 50% for Laboratory A and F, respectively (BDE100 results are shown in Fig. 4 as an example). These intermediate precision values (six measurements spread over 2 days, thus in between repeatability and reproducibility conditions) do not seem to justify estimations of expanded uncertainties up to 50%, especially in the case of Laboratory F. The laboratories might want to revise and optimise a measurement uncertainty that can be adjusted, most probably, significantly downward.

Laboratories seem again more confident (lower uncertainties) in reporting results in fish than in sediment although it might be a more challenging matrix with significantly lower mass fraction levels.

Focusing on the six laboratories participating in both exercises and thus providing results that can be compared for the same analytes across the two studies, the statement is confirmed. Taking the value assignment of BDE100 as a representative example, the laboratories analysing both CRMs report either the same (Laboratories A, E and F) or a significantly lower U (Laboratories B, C and D, submitting two datasets D1 and D2) in the case of the fish analysis (Fig. 4). The laboratories applied the same analytical procedure for the sediment and the fish, with the exception of Laboratories D and E that varied the extraction step. Laboratory D used SPE for the sediment and Soxhlet for the fish, while Laboratory E employed Soxhlet for the sediment and mixed organic solvents for the fish (Ricci et al. 2019).

**Fig. 5:**
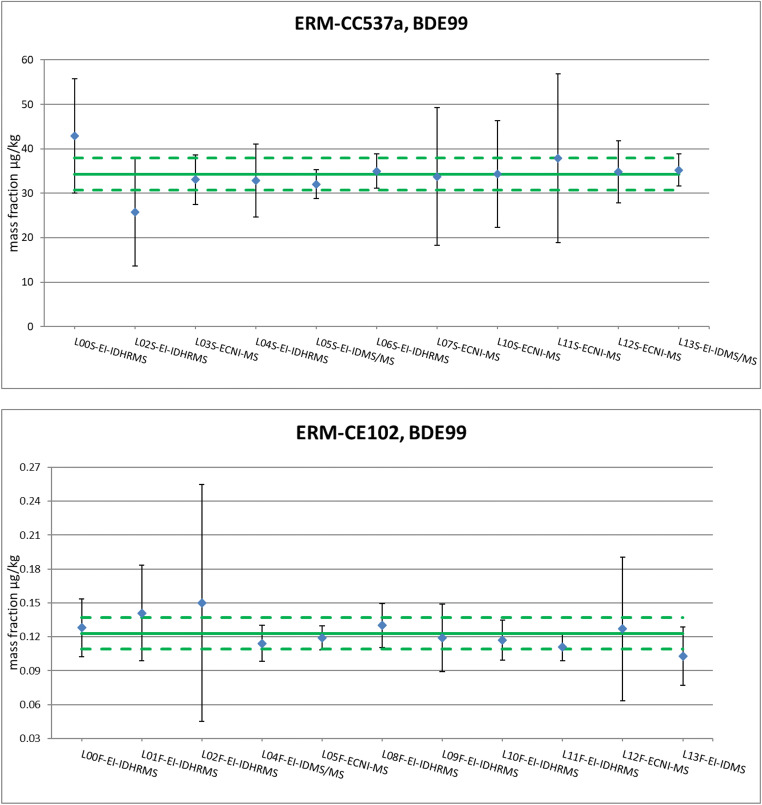
Characterisation datasets of BDE99 in the sediment ERM-CC537a (top) and in the fish ERM-CE102 (bottom). The blue dots are the measurement results and the error bars represent the expanded measurement uncertainty reported by the laboratories; certified value (solid line) ± expanded uncertainty (dashed lines)

An additional consideration can be highlighted about the trueness performance of the laboratories. Considering the certified value as the true value (Fig. 4), most of the laboratories perform very well with regard to trueness; thus, they could avoid in relying on large uncertainties such in the case of Laboratories A and F (but also applicable to Laboratories B and C). Figure 5, showing the characterisation of BDE99 in the two CRMs, confirms this observation: several laboratories could reduce their measurement uncertainty to half and still be close to the certified value. The laboratories’ excellent performance in the characterisation studies of these two CRMs is another reason to encourage them to revisit their uncertainty estimation and to identify contributions which have room to be lowered, to the benefit of the overall measurement uncertainty.

Considering the PBDE results per detection technique applied, there was almost an equal split between the use of GC-ECNI-LRMS (5 laboratories) and GC-EI-ID-HRMS (4 laboratories) for the sediment ERM-CC537a, followed by 3 participants using GC-EI-IDMS/MS and only one using electron ionization with low-resolution MS (GC-EI-ID-LRMS). The picture changes for the characterisation of the fish ERM-CE102 where the vast majority used GC-EI-ID-HRMS (7 laboratories), 2 participants employed GC-ECNI-LRMS and only 1 laboratory relied on either GC-EI-IDMS/MS or GC-EI-ID-LRMS.

Analysing the PBDEs datasets in the characterisation of ERM-CC537a (BDE99 as an example, Fig. 5, top), we can address the extent of the measurement uncertainty with respect to the detection method applied. The use of the isotope dilution for quantification, with ^13^C labelled PBDE internal standards, does not show a consistent reporting of lower uncertainties compared to non-ID techniques. Even when applying the ID approach, there are laboratories declaring uncertainties of up to 50% (and more) for some PBDEs (U = 47%, dataset L02S in Fig. 5, top). It is true that the lowest uncertainties reported (e.g. U = 10%, datasets L05S and L13S and 11% dataset L06S in Fig. 5, top), down to 8%, belong to datasets acquired applying ID, but the use of GC-ECNI-LRMS does not decree per se significantly higher uncertainties. Among the GC-ECNI-LRMS datasets, one laboratory declares U as low as 10–14% for some PBDEs congeners (data not shown, but in Fig. 5, top, the same laboratory, dataset LO3S, reported U = 17%). This finds confirmation in the datasets of the characterisation of ERM-CE102, where another laboratory applying GC-ECNI-LRMS estimated its measurement uncertainty as low as 9% in the determination of BDE99 (dataset L05F in Fig. 5, bottom) and 14% for quantification of BDE100 (Fig. 4, bottom, Lab D2). Beside the low uncertainty reported, it is worth to add here that this laboratory used fluorinated-BDEs as internal standards obtaining very good results also in terms of accuracy (closeness to the certified ‘true’ value).

#### Estimation of reliable measurement uncertainties: the example of the JRC

The outcome of the Key Comparison CCQM-K102 ‘Polybrominated diphenyl ethers in sediment’, coordinated by the JRC and run under the auspices of the Organic Analysis Working Group of the Comité Consultatif pour la Quantité de Matière (CCQM) in 2015, clearly shows the feasibility of lowering the uncertainty budget for this kind of analysis (Ricci et al. 2017). Quantification of BDE47, 99 and 153 at the μg/kg low-middle range in an environmental abiotic matrix (like sediment) was successful for more than 70% of the participating laboratories with expanded measurement uncertainties below 15%.

The JRC participated in the PBDE characterisation of ERM-CC537a, with both GC-ECNI-LRMS (using BDE77 as the internal standard, L12S in Fig. 5) and GC-EI-MS/MS (applying ID with ^13^C-PBDEs, L13S in Fig. 5), as described in the sub-section “Analytical methods”. The two methods were validated in-house using the ERM-CC537a as sample (replicate measurements performed over 5 days) and provided with a full measurement uncertainty budget. The methods were based on the same extraction and clean-up procedures (Shegunova et al. 2017).

The measurement uncertainty was estimated for both methods via a top-down approach based on the validation data, taking into account uncertainty contributions coming from the repeatability, intermediate precision and trueness (Eq. 1 in Online Resource). The uncertainty contribution of the calibration (from the weighing and the purity of the standards used) was for both methods < 0.3%, thus negligible. It should be noted that, to keep this contribution negligible or at least to a minimum, CRMs or standards whose purity is properly assessed should be employed. Repeatability and intermediate precision were estimated using the candidate ERM-CC537a as sample and they span between 1.3 and 12%: during the validation of a method, the use of a reference material can help in keeping these contributions as low as possible. If an in-house QC material is employed, efforts should be made to ensure its homogeneity, so that repeatability contributions are only accounting for the variability of the analytical method and not for any kind of inhomogeneity of the material. The uncertainty contribution of trueness was estimated via a standard addition experiment to a blank material at three different levels. The latter is not the preferable approach, the availability of a fit-for-purpose CRM (with regard to the analyte-matrix combination as well as to the magnitude of the certified value and uncertainty) would ensure a more accurate estimation of the trueness of the method (Linsinger 2019). In this case, it is fundamental that the uncertainty of the CRM is sufficiently small, because it adds up as contribution to the overall trueness uncertainty estimation, with a risk of bringing it to large values.

The uncertainties of the PBDEs measurement results submitted by the JRC for the characterisation of ERM-CC537a are reported in Table 3 (corresponding to six replicates performed over 2 days, as required by the design of the characterisation study). The expanded uncertainties obtained for the GC-EI-IDMS/MS method were generally lower than the ones of the GC-ECNI-LRMS (with the exception of BDE47). It seems that the uncertainties for the GC-ECNI-LRMS method are larger mainly due to higher repeatability and intermediate precision contributions, in the range of 3.3–7.4% and 1.3–12% compared to 3.2–5% and 1.3–4.4% for the GC-EI-IDMS/MS method. Trueness uncertainty contributions are, on the other hand, higher for the GC-EI-IDMS/MS method, spanning between 4 and 6%, while for the GC-ECNI-LRMS, they are between 1 and 4.5%. The trueness uncertainty estimation was carried out for both methods following the same approach of standard addition; thus, it is difficult to understand the reason of such difference.

**Table 3:** Expanded relative uncertainty for the JRC measurement results of PBDEs in the characterisation of ERM-CC537a (6 replicates over 2 days)

BDE	Uncertainty budget %	
	GC-ECNI-LRMS	GC-EI-IDMS/MS
28	9.4	*n.a*.
47	3.7	10.8
100	15.6	11.1
99	20.1	10.4
154	24.1	13.2
153	25.6	13.9
209	*n.a.*	7.8

*n.a.*, not available

These results show that, for the analysis of PBDEs in sediment, uncertainty budgets on average smaller than 20% (depending on the method applied) are attainable and, even when isotope dilution is not available, acceptable uncertainties (< 25%) can be achieved.

## Conclusions

JRC Geel enlarged its portfolio of matrix CRMs for organic contaminants with the successful certification of the sediment ERM-CC537a and the biota ERM-CE102 for brominated flame retardants, to support the work of environmental monitoring and food control laboratories. ERM-CC537a has certified values for the WFD PBDEs and it is the first CRM available for α-, β- and γ-HBCDD diastereoisomers. The fish tissue ERM-CE102 is certified for BDE47, 49, 99, 100, 153 and 154 (while carrying indicative values for BDE28 and 183). The commutability of ERM-CE102 is enhanced by its presentation as a wet paste, with certified values assigned relative to wet weight, directly comparable to established legal limits set on wet weight basis. The mass fractions of the certified BFRs reflect well the levels generally found in routinely analysed samples; thus, these CRMs can be useful for method validation but also as fit-for-purpose quality control tools in the measurement results for regulatory compliance. The in-depth analysis of the characterisation datasets seems to indicate that the general performance of the expert laboratories dealing with the determination of BFRs is satisfactory, with indications of better performances for PBDEs than for HBCDD diastereoisomers. On the other hand, it also highlights that laboratories should aim at a more accurate estimation of their measurement uncertainties. The quality of the measurement results depends not only on the trueness of the data but relies also very much on the confidence level that can be attributed to them.

## Electronic supplementary material

ESM 1 (PDF 1042 kb)
