# Supplementary material for: State of the art in the analysis of brominated flame retardants in biota and sediment: insights from the characterisation of two new certified reference materials
Source: Environ Sci Pollut Res Int. 2020 May 16;28(42):59105–18. doi: 10.1007/s11356-020-08950-7 (PMC8541941; doi:10.1007/s11356-020-08950-7)
Supplement: Supplementary file 1 — (PDF 1042 kb) [file 11356_2020_8950_MOESM1_ESM.pdf]

State of the art in the analysis of brominated flame retardants in biota and sediment: insights from the characterisation of two new certified reference materials.

Marina Ricci [1]*, Penka Shegunova [1], Katrin Vorkamp [2]

[1]European Commission, Joint Research Centre (JRC), Retieseweg 111, 2440 Geel, Belgium

[2]Aarhus University, Department of Environmental Science, Frederiksborgvej 399, 4000 Roskilde, Denmark

* E-mail address: marina.ricci@ec.europa.eu; Tel.: 0032-14-571 962

Online resource - Supplementary Material

Table S1: Summary of methods for PBDEs in the characterisation study of ERM-CC537a

Laboratory code–method	Sample pre-treatment	Detection method<br>Internal standards' details	Type of calibration<br>Calibrants' details (purity)	LOQs<br>[µg/kg dry mass basis]
**L00S-GC-HRMS**	Soxhlet extraction with toluene, clean-up by LLE with conc. H_2SO_4 and multilayer silica column	GC-EI-IDMS<br>MBDE209, MBDE-MXFS (mass labelled PBDE surrogate stock) by Wellington Laboratories (WL)	5 points calibration<br>BDE-CSV-G by WL (solution > 98 %)	0.025 – 0.8
**L01S-GC-MS**	Soxhlet extraction with toluene, clean-up by acidic and basic silica gel and alumina column	GC-EI-IDMS<br>Method 1614 labelled surrogate stock solution by Cambridge Isotope Laboratories (CIL)	6 points calibration<br>ROHS PBDE native PAR spike by CIL (solution ≥ 97.4 %)	0.05 - 5
**L02S-GC-HRMS**	ASE with hexane/acetone 3:1 (v/v), clean-up (except for BDE 209) with silica + alumina and C18-modified silica columns	GC-EI-IDMS<br>BFR-LCS by WL	Single point calibration<br>BFR-PAR, WL (solution > 95 %)	0.002 – 0.01
**L03S-GC-MS**	Soxhlet extraction with hexane/acetone 3:1, clean-up with acidic silica column	GC-ECNI-MS<br>BDE77, BDE128 and ^{13}C-BDE209 by Accustandard and WL	6 points calibration<br>BDE-MXF and BDE209 by WL (solution, purity not specified)	0.05 - 1
**L04S-GC-HRMS**	Soxhlet extraction, clean-up with automatic MIURA system	GC-EI-IDMS<br>Single congener ^{13}C labelled standard solutions by CIL	5 points calibration<br>Single congener native standards by CIL (solutions > 98 %)	0.01

**L05S-GC-MS/MS**	Solid phase extraction (SPE) with MeOH and hexane, clean-up with multilayer silica column and Cu	GC-EI IDMS/MS<br><br>^{13}C labelled BDE28, 47, 99, 153,183, 209 by WL	2 points bracketing calibration<br><br>Single congener native standards by Accustandard and WL (solutions, purity not specified)	0.1<br><br>15 (BDE 209)
**L06S-GC-HRMS**	Soxhlet extraction with toluene, clean-up by adsorption chromatography	GC-EI-IDMS<br><br>Single congener ^{13}C labelled standard solutions by CIL	Single point calibration<br><br>Single congener native standards by CIL<br>(solutions > 98 %)	0.002
**L07S-GC-MS**	Soxhlet extraction with pentane/CH_2Cl_2, clean-up with acidic silica column	GC-ECNI-MS<br><br>BDE58 and ^{13}C-BDE209 by Accustandard and CIL	10 points calibration<br><br>NIST SRM 2257 and 2258 (certified solutions)	0.03 – 0.8
**L09S-GC-HRMS**	Extraction on a Büchi automated extraction system with toluene, clean-up with multilayer silica column	GC-EI-IDMS/MS<br><br>BFR-LCS-STK (^{13}C labelled BDEs stock standard) by WL	10 points calibration<br><br>BFR-CVS by WL<br>(solution > 98 %)	not reported
**L10S-GC-MS**	ASE with hexane-acetone 3:1 (v/v), clean-up with acidic silica column and gel permeation chromatography	GC-ECNI-MS<br><br>BDE58 and ^{13}C-BDE209 by WL	8 points calibration<br><br>BDE-MXE by WL<br>(solution, 1 to 5 µg/mL ± 5 %)	0.1-0.3
**L11S-GC-MS**	Soxhlet extraction (adding of Cu) with hexane/acetone 4/1, clean-up with alumina and acidic silica column	GC-ECNI-MS<br><br>^{13}C-Polychlorinated naphthalenes and ^{13}C-BDE209 by CIL	10 points calibration<br><br>Single congener native standards by CIL<br>(solutions > 98 %)	0.05-0.15
**L12S-GC-MS**	ASE hexane/acetone, 3:1 with Cu/Na_2SO_4, clean-up by SPE	GC-ECNI-MS<br><br>BDE77 by Accustandard	6 points calibration<br><br>NIST SRM 2257<br>(certified solution)	0.1-1.2
**L13S-GC-MS/MS**	ASE hexane/acetone 3:1 with Cu/Na_2SO_4, clean-up by SPE	GC-EI-IDMS/MS<br><br>Single congener ^{13}C labelled standard solutions by CAMPRO	6 points calibration<br><br>Single congener native standards by Chiron<br>(solutions > 97.5 %, except BDE209 > 95 %)	0.04-10.7
**Not used in certification**				
**L08S-GC- MS/MS**	Soxhlet extraction (adding of Cu filings), clean-up with deactivated alumina column	GC-EI-MS/MS<br><br>F-BDE69 and F-BDE60	10 points calibration<br><br>BDE-MXE by WL<br>(solution, purity not specified)	0.1, 0.2

Instrumental analysis methods applied by the JRC for the analysis of PBDEs in the characterisation study of ERM-CC537a

GC-ECNI-MS (L12S, Table S1)*:* Agilent 5973 MSD, 6890 GC (Agilent Technologies, Santa Clara, CA, USA), Chemstation software, column DB-5HT 15 m*0.25 mm*0.15 µm (Agilent Technologies, Santa Clara, CA, USA), PTV injector 90 °C, oven (program): 90 °C (1.5 min), 20 °C /min to 270 °C, 10 °C /min to 285 °C , 35 °C /min to 300 °C (7 min), 50 °C /min to 320 °C (5 min), transfer line 250 °C, carrier gas helium, injection volume 1 µL.

GC-EI-IDMS/MS (L13S, Table S1)*:* Thermo Scientific, TSQ Quantium XLS Ultra Trace 1310 (Thermo Fisher Scientific, Austin,TX, USA), XCalibur software, column DB-5HT 15 m*0.25 mm*0.10 µm (Agilent Technologies, Santa Clara, CA, USA), Split/Splitless (SSL) injector 120 °C, oven (program): 120 °C (2 min), 15 °C /min to 230 °C, 5 °C /min to 270 °C , 10 °C /min to 330 °C (5 min), transfer line 270 °C, carrier gas helium, injection volume 2 µL.

Measurement uncertainty for PBDEs in sediment

The formula applied for the estimation of the expanded measurement uncertainty (U) in the determination of PBDEs in sediment in the two in-house validated methods is the following:

$$U = k * \sqrt{\frac{RSD_{rep}^2}{n_1} + \frac{RSD_{ip}^2}{n_2} + u_{true}^2 + u_{cal}^2} \qquad \textbf{Eq. 1}$$

k coverage factor (k=2) resulting in a confidence level of approximately 95 %
RSD_{rep} – relative standard deviation of repeatability, %
RSD_{ip} – relative standard deviation of intermediate precision, %
n_1 – number of replicates
n_2 – number of days
u_{true} – uncertainty of trueness estimation, %
u_{cal} – uncertainty of the preparation of the calibration solutions, %

Table S2: Summary of methods for HBCDDs in the characterisation study of ERM-CC537a

Laboratory code–method	Sample pre-treatment	Detection method<br>Internal standard(s) details	Type of calibration<br>Calibrants' details (purity)	LOQ<br>[µg/kg dry mass basis]
**L03S-LC-MS/MS**	Soxhlet extraction with hexane/acetone, clean-up with acidic silica	HPLC-ESI negative-IDMS/MS<br>Single isomers ^{13}C-HBCDD by WL	6 points calibration<br>Single isomers HBCDD by WL (solution > 98 %)	0.5
**L04S-LC-MS/MS**	QuEChERS extraction	HPLC-ESI negative-IDMS/MS<br>Single isomers ^{13}C-HBCDD by WL	10 points calibration<br>Single isomers HBCDD by WL (solutions, purity not specified)	0.2
**L05S-LC-MS/MS**	Solid phase extraction (SPE) with MeOH and hexane	UPLC-ESI negative-IDMS/MS<br>^{13}C-γ-HBCDD	7 points calibration<br>Single isomers HBCDD by WL (solutions, purity not specified)	0.3
**L06S-LC-MS/MS**	Extraction by sonication in CH_2Cl_2, clean-up by adsorption chromatography	UPLC-ESI negative-IDMS/MS<br>Single isomers ^{13}C-HBCDD by WL	4 points calibration<br>Single isomers HBCDD by WL (solution > 98 %)	0.2
**L10S-LC- MS/MS**	ASE with hexane-acetone 3:1 (v/v), clean-up with acidic silica column and gel permeation chromatography	HPLC-ESI negative-IDMS/MS<br>Single isomers ^{13}C-HBCDD by WL	8 points calibration<br>Single isomers HBCDD by WL (solutions, purity not specified)	0.1
**L11S- LC-MS/MS**	Soxhlet extraction (adding of Cu) with hexane/acetone 4/1, clean-up with alumina and acidic silica column	HPLC-ESI negative-IDMS/MS<br>Single isomers ^{13}C-HBCDD by CIL	10 points calibration<br>Single isomers HBCDD by CIL (solutions: α- and β-HBCDD > 98 %, γ-HBCDD > 97 %)	0.05
**Not used in certification**				
**L00S-LC-MS/MS**	Extraction by shaking with MeOH/ACN, centrifugation, dilution 1:1 with milliQ water	UPLC-ESI-IDMS/MS<br>Deuterium labelled γ-HBCDD	8 points calibration<br>Single isomer standards by WL (solution, > 98 %)	0.8 - 1.5
**L07S-LC-MS**	Soxhlet extraction, clean-up with sulfuric acid	HPLC-ESI negative-IDMS<br>Single isomers ^{13}C-HBCDD	7 points calibration<br>Single isomer standards by CIL (solutions, purity not specified)	1
**L08S-UPLC-MS/MS**	Soxhlet extraction (adding of Cu filings), clean-up with gel permeation chromatography and acidified silica column	UPLC-ESI-IDMS/MS<br>Deuterium labelled single isomers HBCDD by WL	7 points calibration<br>Single isomer standards by WL (solution > 98 %)	0.75

Table S3: Summary of methods for PBDEs in the characterisation study of ERM-CE102

Laboratory code–method	Sample pre-treatment	Quantification method<br>GC column<br>Internal standard(s)	Calibration and calibrants' details (purity)	LOQs [ng/kg wet weight]
**L00F-GC-HRMS**	Sample added with water, labelled internal standards (i.s.) and EtOH. Consecutive extractions with diethylether and *n*-pentane, after separation the organic phase was treated with conc. H_2SO_4, re-extracted with *n*-hexane and cleaned-up on a multilayer silica gel column containing acidic, basic and neutral silica.	GC-EI-IDHRMS<br>RTX-5 Sil MS 30 m x 0.25 mm x 0.25 µm<br>MBDE-MXE (^{13}C labelled PBDE solution) by Wellington Laboratories (WL)	8 points from 0.5 to 500 pg for Tri–PeBDE<br>8 points from 1 to 2500 pg for Hx–HpBDE<br>BDE-MXE, WL (> 98 %)	TriBDE <0.5<br>TeBDE <1<br>PeBDE 1 - 2<br>HxBDE 2 - 3<br>HpBDE 2- 3
**L01F-GC-HRMS**	After spiking with labelled i.s and adding Na_2SO_4, Soxhlet extraction with hexane/CH_2Cl_2, clean-up by liquid-liquid extraction with conc. H_2SO_4 and multilayer silica gel column (EPA Method 1614A)	GC-EI-IDHRMS<br>STX 500, 11 m, 0.25 mm ID, 0.15 µm<br>MBDE-MXFS MXE (13C labelled PBDE solution) by WL	5 points, 1-400 ng/ml; 5-2000 ng/ml<br>BDE-CSV-G by WL (≥ 98 %)	5-10
**L02F-GC-HRMS**	After spiking with labelled i.s., extraction with 10 % CH_2Cl_2/hexane on Automatic Pressurized Extraction Unit, clean-up via Power Prep System - 3 columns - silica, alumina and carbon (Method E3481- MECP)	GC-EI-IDHRMS<br>J&W DB-5HT, 15 m x 0.25 mm x 0.1 µm<br>$^{13}C_{12}$ - Labelled BDE Standard, BFR-LCS by WL	4 points<br>BFR-BDE-CVS, WL	BDE28 : 5.2<br>BDE47: 311<br>BDE49: 6.1<br>BDE99: 217<br>BDE100: 40.6<br>BDE153: 10.4<br>BDE154: 8.7<br>BDE183: 9.1

**L04F-GC-MS/MS**	After spiking with labelled i.s., Soxhlet extraction with hexane:acetone, clean-up on a multilayer silica gel column containing acidic, basic and neutral silica	GC-EI-IDMS/MS<br>Rtx1614, 15 m x 0,25 mm ID x 0.1 µm<br>Single congener ^{13}C labelled standard solutions by WL	2 points: 1000 and 2000 pg/L (linearity tested with 10 points)<br>Single congener native standards by WL (> 98 %)	3 for all BDEs except BDE183: 4
**L05F-GC-MS**	After spiking with i.s., Soxhlet extraction with hexane:acetone, clean-up on a multilayer silica gel column containing acidic, basic and neutral silica	GC-ECNI-MS<br>Rtx1614, 15 m x 0.25 mm ID x 0.1 µm<br>F-BDE28, 47, 99, 160 by Chiron	2 points: 1000 and 2000 pg/L (linearity tested with 10 points)<br>Single congener native standards by WL (> 98 %)	BDE28 : 3<br>BDE47, 153, 154, 183: 13<br>BDE49: 8<br>BDE99: 7<br>BDE100: 6
**L08F-GC-HRMS**	After spiking with labelled i.s., Soxhlet extraction with toluene and water separator followed by Soxhlet extraction with toluene:EtOH 1:2, clean-up with automatic MIURA system (silvernitrate silica, sulfuric acidic silica, carbon and alumina columns)	GC-EI-IDHRMS<br>Phenomenex SemiVolatiles, L: 20 m, ID: 0.18 mm, FT: 0.18 µm<br>Single congener ^{13}C labelled standard solutions by Cambridge Isotope Laboratories (CIL)	6 points: 1, 5, 25,100, 500, 2500 pg/5µL<br>Single congener native standards by CIL (> 98 %)	BDE28 : 1<br>BDE47: 20<br>BDE49: 2<br>BDE99: 15<br>BDE100: 4<br>BDE153, 154: 5<br>BDE183: 3
**L09F-GC-HRMS**	After spiking with labelled i.s., extraction by EtOAc, (purification by dispersive acidified silica clean-up for the lipid-rich samples), clean-up by silica column chromatography (1 gram activated silica and 8 gram acidified silica)	GC-EI-IDHRMS<br>Rtx-ClPesticides, 30 m x 0.25 mm i.d. x 0.25 mm<br>mix of ^{13}C-labelled BDEs, company not specified	9 points: 0, 50, 200, 500, 2000, 5000, 20000, 50000, 100000 pg/mL<br>PBDE mix 10, S-4559-50-T (50 µg/ml +/- 5 %)	< 1

**L10F-GC-HRMS**	After spiking with labelled i.s., ASE extraction with *n*-hexane/acetone 3:1, clean-up with an automated system comprising 3 chromatographic columns: multilayer (SiO_2 + SiO_2 with H_2SO_4), alumina, carbon	GC-EI-IDHRMS<br>Rtx-1614, 15 m, ID 0.25 mm, 0.1 µm<br>$^{13}C_{12}$ - Labelled BDE Standard, BRF-LCS by WL	Single point<br>BFR-PAR native compounds stock solution by WL (purity relative uncertainty ± 5 %)	BDE28: 0.8<br>BDE47: 15<br>BDE99: 5.5<br>BDE100: 1.3<br>BDE153: 5.4<br>BDE154: 1.2<br>BDE183: 2.0
**L11F-GC-HRMS**	After spiking with labelled i.s., exhaustive extraction with mixed organic solvents, clean-up by adsorption chromatography and further on modified silica and alumina	GC-EI-IDHRMS<br>Rtx-1614 30 m x 0.25 mm ID x 0.1 µm<br>MBDE-MXE (^{13}C labelled PBDE solution) by WL and SCFB-004 by CIL	Single point<br>ROHS PBDE Native PAR Spike by CIL<br>(> 98 %)	2-3
**L12F-GC-MS**	Soxhlet extraction with hexane:acetone (4:1), column clean-up on aluminium oxide, silica and silica with sulphuric acid	GC-ECNI-MS<br>J&W DB-5, 60 m, 0.25 mm, 0.25 µm<br>BDE71	8 points, range: 0.05-10 ng/mL<br>Single congener native standards by CIL<br>(> 98 %)	5 for all BDEs except<br>BDE183: 10-11
**L13F-GC-MS**	After spiking with labelled i.s., Soxhlet extraction with of *n*-hexane:CH_2Cl_2, clean-up with H_2SO_4-Si column followed by a second alumina oxide column	GC-EI-IDMS<br>RTX 1614 15 m, 0.10 µm film thickness, 0.25 mm ID<br>Single congener ^{13}C labelled standard solutions by WL	5 points<br>Single congener native standard solutions by WL<br>(> 98 %)	BDE28, 47, 49: 1<br>BDE99, 100: 2<br>BDE153, 154: 3<br>BDE183: 5

Table S4: Results of the homogeneity evaluation

u_{bb} = homogeneity uncertainty

	ERM-CC537a	ERM-CE102
	$u_{bb,rel}$ [%]	
BDE28	1.9	0.6
BDE47	1.6	0.2
BDE49	--	9.3
BDE99	2.3	0.5
BDE100	1.9	0.6
BDE153	3.8	0.6
BDE154	3.1	0.6
BDE183	5.2	1.2
BDE209	1.0	24.4
α-HBCD	3.6	--
β-HBCD	4.1	--
γ -HBCD	3.9	--

Table S5: Results of the stability evaluation

	ERM-CC537a		ERM-CE102	
	$u_{sts,rel}$ [%]	$u_{lts,rel}$ [%]	$u_{sts,rel}$ [%]	$u_{lts,rel}$ [%]
BDE28	1.1	6.1	0.6	3.8
BDE47	0.9	2.9	0.2	2.8
BDE49	--	--	--	5.3
BDE99	0.5	3.0	0.3	4.1
BDE100	0.8	3.3	0.7	2.4
BDE153	0.7	3.2	0.3	4.2
BDE154	0.7	3.9	0.3	2.5
BDE-183	0.7	3.2	0.7	4.0
BDE209	0.9	2.0	--	--
α-HBCD	1.1	5.5	--	--
β-HBCD	1.2	5.2	--	--
γ-HBCD	1.0	9.2	--	--

The uncertainties of stability during dispatch (short-term stability, $u_{sts,rel}$) were calculated for a temperature of 18 °C and 1 week; the uncertainties of stability during storage (long-term stability, $u_{lts,rel}$) were calculated for a storage temperature of 18 °C and 18 months for ERM-CC537a and for a storage temperature of 4 °C and 24 months for ERM-CE102.

Table S6: Certified values and uncertainties (k=2) for ERM-CC537a and for ERM-CE102 expressed in µg/kg (if not otherwise noted); in italic indicative values

	**ERM-CC537a** **µg/kg** (dry mass)		**ERM-CE102** **µg/kg** (wet weight)	
	Certified value	U_{CRM}	Certified value	UCRM
BDE28	0.28	0.05	*0.0077*	*0.0010*
BDE47	16.5	1.8	0.227	0.019
BDE49	--	--	0.033	0.008
BDE99	34	4	0.124	0.014
BDE100	5.8	0.6	0.060	0.006
BDE153	6.6	0.9	0.069	0.007
BDE154	3.5	0.5	0.110	0.008
BDE183	1.41	0.21	*0.014*	*0.004*[1)]
BDE209	7.8 mg/kg	0.7 mg/kg		
α-HBCD	8.3	1.6		
β-HBCD	2.3	0.5		
γ-HBCD	60	16		

[1)] k=2.571

Table S7: Uncertainty of characterisation for ERM-CC537a and ERM-CE102

	ERM-CC537a	ERM-CE102
	$u_{char,rel}$ [%]	
BDE28	5.4	4.8
BDE47	4.1	3.1
BDE49	--	4.3
BDE99	3.6	3.3
BDE100	2.9	3.6
BDE153	4.3	2.5
BDE154	3.9	2.6
BDE183	4.1	8.8
BDE209	3.6	--
α-HBCD	6.7	--
β-HBCD	7.1	--
γ -HBCD	8.2	--
